# Alveolar Rhabdomyosarcoma of Nasopharynx and Paranasal Sinuses in Children Diagnosis and Treatment—Review of the Literature and Case Report

**DOI:** 10.3390/medicina61010080

**Published:** 2025-01-06

**Authors:** Cristian Mârțu, Ștefan Dragoș Tîrnovanu, Ioana Mârțu, Dan Ferariu, Dan Mârțu, Alexandra Jităreanu, Luminița Rădulescu

**Affiliations:** 1 ENT Clinic Department, “Grigore T. Popa” University of Medicine and Pharmacy Iasi, Universitatii Street 16, 700115 Iasi, Romania; martu.cristian@umfiasi.ro (C.M.); dvmartu@yahoo.com (D.M.); luminita.radulescu@umfiasi.ro (L.R.); 2 Department of Orthopedics and Traumatology, Faculty of Medicine, “Grigore T. Popa” University of Medicine and Pharmacy, 700115 Iasi, Romania; stefan-dragos.tirnovanu@umfiasi.ro; 3 Department of Orthopaedics and Traumatology, “Sf. Spiridon” Emergency Universitary Hospital, 700115 Iasi, Romania; 4 Department of Oral Implantology, Removable Dentures and Technology, “Grigore T. Popa” University of Medicine and Pharmacy Iasi, Universitatii Street 16, 700115 Iasi, Romania; 5 Department of Pathology, Regional Institute of Oncology, 700483 Iasi, Romania; d_ferariu@yahoo.com; 6 Department of Toxicology, Faculty of Pharmacy, “Grigore T. Popa” University of Medicine and Pharmacy, 700115 Iasi, Romania; jitareanu.alexandra@umfiasi.ro

**Keywords:** alveolar rhabdomyosarcoma, rhinopharynx, paranasal sinuses, pediatric

## Abstract

Alveolar rhabdomyosarcoma (aRMS) is a rare pediatric malignant tumor with a poor prognosis, particularly when located in the rhinopharynx and sphenoidal floor, which complicates diagnosis and increases the risk of misclassification as benign growths. The specific genotype of aRMS is associated with a worse clinical outcome. In young children, especially those aged 4 to 12 years, rhinopharyngeal masses are often attributed to chronic adenoiditis; however, other benign (e.g., angiofibroma in boys) and malignant tumors may also be present. Initial symptoms frequently include nasal obstruction, muco-purulent nasal discharge, serous otitis media, sinusitis, and epistaxis. Rhabdomyosarcoma is the second most common ENT neoplasm in children, following lymphoma, with an incidence of approximately 6 cases per 1,000,000 annually. This report presents the case of an 8-year-old boy diagnosed with aRMS, accompanied by a literature review. Alveolar rhabdomyosarcoma should be suspected in children presenting with a vegetative tumor in the rhinopharynx or paranasal sinuses. Combined treatment approaches (surgery, radiotherapy, and chemotherapy) should be tailored to tumor characteristics. Neuronavigation-guided functional endoscopic sinus surgery (FESS) is an effective option for achieving complete tumor excision, depending on tumor size and extent. The prognosis remains reserved and is contingent upon accurate evaluation and timely intervention. Rigorous follow-up, including endoscopic and imagistic investigation, is crucial for early detection of recurrences, thereby improving treatment outcomes.

## 1. Introduction

Rhabdomyosarcoma (RMS) is a malignant tumor composed of rhabdomyoblasts, which microscopically resemble striated muscle cells [1,2,3]. First described by Weber in 1854 and cited by Ruymann [4], a more comprehensive characterization of pediatric RMS was provided by Pack in 1952 [5]. Alveolar RMS (aRMS) is a common pediatric soft tissue malignancy linked to a chromosomal translocation that results in the expression of a PAX3/7-FOXO1 fusion protein [6]. However, alternative genetic associations have also been documented [7].

Pediatric neoplasms comprise approximately 5% of all cancers in children and are the second leading cause of mortality in those aged 5–14 years [8], following accidental deaths [9]. Annually, 11,000 new cases are diagnosed in individuals under 20 years of age [8]. Among pediatric neoplasms [10], lymphomas are the most prevalent (59%), followed by RMS (13%), rhinopharyngeal cancer (5%), neuroblastoma (5%), soft tissue sarcoma (4.5%), salivary gland tumors (2.5%), and malignant teratomas [8].

RMS represents the most common soft tissue sarcoma in children, accounting for 4.5% of pediatric cancers [11,12], with an incidence of 6 cases per 1,000,000 annually [11]. The most frequently affected sites are the head and neck, genitourinary tract, and extremities [13]. Most cases are diagnosed by age 10, with a male-to-female ratio of 1.5:1 [8]. Head and neck RMS constitute approximately 40% of all RMS cases, and one-third of the cases involving the cephalic extremity invade the orbit [14]. Rare cases have also been reported of RMS localized to the skin of the nasal pyramid [15,16].

Additional case reports further illustrate the complexity of managing RMS in the sinonasal region. For example, a case documented by Liu et al. reported alveolar RMS metastasizing to the breast, highlighting the aggressive nature of the disease [17]. Another case by Watanapokasin et al. presented an unusual case of bilateral proptosis due to adult-onset alveolar RMS of the ethmoid sinus [18].

## 2. RMS Etiology

The etiology of RMS remains unclear, although genetic predisposition may be a contributing factor. Genetic anomalies associated with aRMS correlate with poorer clinical outcomes, highlighting the importance of genetic evaluation in guiding treatment strategies [19,20]. Li-Fraumeni syndrome, an autosomal dominant disorder associated with germline p53 mutations, is among the risk factors; affected individuals often have a family history of various carcinomas, particularly premenopausal breast cancer. Other cited risk factors include neurofibromatosis, radiation exposure, and maternal use of substances such as cocaine, marijuana, and alcohol during pregnancy [21].

## 3. RMS Classification

The World Health Organization (WHO) has recently updated staging and classification guidelines for rhabdomyosarcoma (RMS) [22]. Rhabdomyosarcoma (RMS) staging has been updated to incorporate advances in molecular diagnostics and a refined understanding of tumor biology, enabling more accurate risk stratification and personalized treatment. The WHO classification emphasizes the importance of detecting specific gene fusions, such as PAX3-FOXO1 in ARMS and NCOA2 rearrangements in spindle cell variants, to help differentiate RMS subtypes and predict patient outcomes. Genetic profiling is crucial for assessing the prognosis of RMS. For example, tumors with MYOD1 mutations or PAX-FOXO1 fusions tend to have a worse prognosis, while those with VGLL2 rearrangements in the spindle cell subtype often exhibit better clinical outcomes.

The World Health Organization (WHO) classification of rhabdomyosarcoma (RMS) has evolved to include distinct subtypes with updated genetic insights and clinical relevance [22]. The WHO classification now categorizes RMS into several types based on both histological and molecular features:

1. Embryonal Rhabdomyosarcoma (ERMS): This is the most common subtype, primarily affecting children. ERMS can be present in various forms, including botryoid and diffuse types. The botryoid variant has a favorable prognosis, especially when it arises in mucosal-lined sites such as the bladder or vagina.
2. Alveolar Rhabdomyosarcoma (ARMS): Characterized by PAX3-FOXO1 or PAX7-FOXO1 gene fusions, ARMS is more aggressive than ERMS and is typically found in older children or adolescents. These tumors often have a worse prognosis due to the presence of these fusion genes, which disrupt normal cellular differentiation.
3. Spindle Cell/Sclerosing Rhabdomyosarcoma: This subtype is rare and is noted for its spindle-shaped cells and sclerotic background. It is most commonly found in the head and neck or extremities, with a predilection for the paratesticular region in pediatric patients.
  Spindle cell RMS can be further categorized based on genetic findings:
  - VGLL2 and NCOA2 rearrangements are typically seen in infantile cases, which tend to have a favorable prognosis.
  - MYOD1 mutations, more common in adolescents and adults, are associated with a poor prognosis.
  - The intraosseous variant, involving TFCP2::NCOA2 fusion genes, is an additional form that can be identified in bone tissues.
4. Pleomorphic Rhabdomyosarcoma: This subtype, mainly seen in adults, is characterized by pleomorphic tumor cells (irregular shapes and sizes). Pleomorphic RMS is associated with a poor prognosis due to its more aggressive clinical behavior [22].

1. Embryonal RMS
  - Description: Characterized by primitive, ovoid, or angulated spindle-shaped cells.
  - Histology: Scattered rhabdomyoblasts (cells showing skeletal muscle differentiation).
  - Variant: A subset may exhibit diffuse anaplasia, which is associated with worse prognosis.
  - Significance: It is the most common subtype in children and has a relatively favorable prognosis.
2. Alveolar RMS
  - Description: Composed of monotonous round cells.
  - Histology: May display a distinct alveolar pattern, resembling small clusters of cells in spaces (“alveoli”).
  - Genetics: Often associated with fusion genes, such as PAX3-FOXO1 or PAX7-FOXO1, linked to a more aggressive course.
  - Significance: Typically seen in adolescents and young adults.
3. Spindle Cell RMS of MYOD1-mutated Type
  - Description: Features fascicles of spindle cells with areas of primitive round cells.
  - Histology: Dense sclerosis may be present, creating a pseudoalveolar pattern. Rare rhabdomyoblasts may be observed.
  - Genetics: Associated with MYOD1 mutations, which carry a poor prognosis.
  - Significance: This subtype differs from classic spindle cell RMS due to its molecular profile and aggressive behavior.
4. Osseous Spindle Cell RMS
  - Description: Histology ranges from mixed spindled to epithelioid, with less frequent pure spindled or epithelioid patterns.
  - Significance: This rare subtype highlights the morphological diversity of RMS, which can include areas mimicking bone formation.
5. Fusion-Driven Spindle Cell RMS
  - Description: A heterogeneous group with variable spindle cell morphology.
  - Genetics: Defined by specific gene fusions, which influence its clinical and pathological behavior.
  - Significance: This emerging category underscores the importance of molecular studies in diagnosing RMS.
6. Spindle Cell RMS, NOS (Not Otherwise Specified)
  - Description: Appears as a non-descript spindle cell sarcoma with rhabdomyoblastic differentiation.
  - Significance: Lack of distinct genetic or histological markers places it in this catch-all category.
7. Pleomorphic RMS
  - Description: Composed of large, pleomorphic rhabdomyoblasts.
  - Histology: Displays high-grade features with significant cellular atypia and pleomorphism.
  - Significance: Predominantly occurs in adults and has a poor prognosis.
8. Epithelioid RMS
  - Description: Features a distinct epithelioid morphology resembling carcinoma or melanoma.
  - Histology: A unique presentation of RMS with cells mimicking epithelial tumors.
  - Significance: Highlights the potential diagnostic challenges due to their resemblance to non-sarcomatous malignancies.
9. RMS Arising in Immature Rhabdomyoblastic Tumors (IRMT)
  - Description: Morphology ranges from epithelioid to rhabdoid or undifferentiated spindle to epithelioid cells.
  - Significance: Reflects RMS transformation in a previously identified precursor tumor type, potentially impacting clinical management.

This classification system captures the morphological diversity of RMS and integrates molecular features, aiding in tailored diagnostic and therapeutic approaches [22].

## 4. RMS in Differential Diagnoses

Among the various subtypes, alveolar RMS (ARMS) represents a significant clinical challenge due to its aggressive nature and potential for local invasion and metastasis [23,24]. In adults, RMS commonly arises in the deep soft tissues; however, it can originate in atypical sites such as the nasal cavity and paranasal sinuses, presenting unique diagnostic and therapeutic challenges [25,26].

The presentation of the nasal cavity or paranasal sinus RMS can be subtle and may mimic more common pathologies. Symptoms may include nasal obstruction, epistaxis, or facial swelling, which can delay diagnosis [24,26].

The presence of a tumor-like mass in the nasal region necessitates a comprehensive clinical and paraclinical evaluation to ensure optimal treatment outcomes [13]. Differential diagnoses must include various benign tumors such as papilloma, fibromyoma, chondroma, adenoma, glioma, neuroblastoma, and angiofibroma. Rapid growth and imaging studies, particularly CT scans showing invasion of adjacent structures, can help exclude many benign conditions. In pediatric cases, lymphoma is the most common malignant tumor affecting the nasal fossa.

Imaging studies, particularly MRI and CT scans, are crucial for assessing tumor extent and involvement of surrounding structures [17,27].

Computed Tomography (CT) is the initial imaging modality for patients with sinonasal pathology, particularly when evaluating persistent symptoms. It is indispensable for assessing bony structures, such as cortical destruction, remodeling, and sclerosis, associated with sinonasal malignancies. CT imaging is particularly effective for identifying aggressive bone destruction patterns (as in squamous cell carcinoma). In sinonasal lymphomas, CT reveals lobulated, hyperdense masses with variable bony involvement [28].

MRI excels in evaluating soft tissue details and tumor extensions, particularly into the skull base or intracranial structures. With superior contrast resolution, MRI helps differentiate tumor tissues from inflammation or post-obstructive secretions. Techniques like diffusion-weighted imaging (DWI) and apparent diffusion coefficient (ADC) mapping enhance MRI’s ability to distinguish between benign and malignant sinonasal conditions. For instance, ADC values can help separate lymphomas and SCC from benign lesions [28].

Together, CT, MRI, and PET/CT offer a comprehensive tumor evaluation. While CT defines bone involvement and MRI highlights soft tissue characteristics, PET/CT assesses tumor metabolism, aiding in diagnosis, staging, and surveillance. This multimodality strategy enhances diagnostic accuracy and informs tailored treatment plans for sinonasal malignancies [29].

MRI plays a complementary role to CT, especially in specific clinical scenarios. It is advantageous for staging sinonasal masses, assessing anosmia, and identifying complications of sinusitis. MRI is particularly beneficial in imaging niche anatomical areas, such as the skull base, nasal vestibule, and olfactory niches, where CT may not provide adequate soft tissue detail [29].

On CT, sinonasal SCC appears as a soft tissue mass with bone destruction. MRI shows T1 iso intensity, T2 hypointensity, and variable enhancement, with larger tumors exhibiting heterogeneous signals due to necrosis or hemorrhage [30]. SCC often invades the skull base and cranial fossae and is best seen on contrast-enhanced, fat-suppressed T1 MRI [31]. Moderate to intense FDG uptake on 18F-FDG PET/CT supports diagnosis and staging [32].

Olfactory neuroblastoma (ONB) typically involves the cribriform plate, often with intracranial extension and a dumbbell shape. CT shows an expansile mass with bone erosion and occasional speckled calcifications or hyperostosis [33]. MRI reveals low T1 and high T2 signals with homogeneous or heterogeneous enhancement. Flow voids indicate high vascularity, and cystic changes may appear at the superior margins [30]. 18F-FDG PET/CT aids in detecting metastases and monitoring but shows no correlation between tumor grade and SUVmax [34].

Sinonasal adenocarcinoma shows isointense signals on T1-weighted MRI (T1WI) and variable T2-weighted MRI (T2WI) signals, depending on cellularity: high in paracellular tumors and intermediate in cellular ones [30]. Unlike SCC, which favors the maxillary antrum and nasal cavity, it typically originates in the ethmoid sinuses. Adenocarcinomas have more defined margins and muscular enhancement than SCCs [35]. 18F-FDG PET/CT is useful for staging and detecting recurrences, helping differentiate tumors from postoperative changes [36].

Primary sinonasal malignant melanoma (SNM) commonly appears as a well-defined polypoid mass on CT with smoothly marginated bony remodeling despite its aggressive nature. Contrast enhancement is variable, and melanin often causes T1 shortening, complicating characterization [30].

Amelanotic SNMs show low signal intensity on T1- and T2-weighted MRI [37]. Intense FDG uptake is typically observed on 18F-FDG PET/CT, especially in cases of recurrence or residual disease [38].

On non-contrast CT, sinonasal lymphomas appear as lobulated, slightly hyperdense masses with bone destruction or remodeling. Calcifications are rare, thin, and linear, typically reflecting sinus wall fragments rather than tumor calcification involvement. Contrast-enhanced CT highlights high cellularity with homogeneous enhancement, while necrosis is uncommon [30].

MRI shows isointense T1 signals with homogeneous enhancement and hypointense T2/STIR signals due to high cellularity. Low ADC values on DWI further reflect this feature [39]. Intense FDG uptake is a characteristic finding in PET imaging [40].

Sinonasal undifferentiated carcinoma (SNUC) typically appears as a large, destructive mass with ill-defined margins, bone destruction, and invasion of adjacent structures. CT imaging reveals irregular borders, aggressive bone destruction, and variable enhancement without calcifications [30]. On MRI, the tumor is isointense to muscle on T1WI and iso- to hyperintense on T2WI, with heterogeneous contrast enhancement [41].

SNUC demonstrates a significantly higher SUVmax on 18F-FDG PET/CT than esthesioneuroblastoma (ENB), reflecting its increased metabolic activity and aggressiveness.

Adenoid cystic carcinoma (ACC) commonly affects the maxillary antrum and nasal cavity, with a hallmark tendency for perineural spread, often involving trigeminal nerve branches in the pterygopalatine fossa. Lower-grade ACCs show homogeneous enhancement and bone remodeling, while higher-grade tumors display necrosis and destruction. MRI typically reveals intermediate T2 signal intensity, with low-grade tumors occasionally mimicking inflammatory tissue due to high T2 signal. Neural foraminal enlargement and abnormal perineural signals are key indicators of perineural invasion [30]. 18F-FDG PET/CT has limited utility due to ACC’s low metabolic activity [42].

Chondrosarcoma, the most common sinonasal sarcoma, typically arises in the nasal septum and extends along the ethmoid bone to the skull base and palate [43]. CT shows a lobulated, heterogeneous mass with stippled chondroid calcifications and hypodensity due to the chondroid matrix [Articol Cristi]. MRI reveals a hyperintense T2 signal, hypointense T1 signal, and signal voids from calcifications. Contrast-enhanced imaging highlights a heterogeneous enhancement pattern, with the avascular chondroid matrix remaining unchanged [44].

Osteosarcomas in the sinonasal region may be primary or extend from adjacent areas. CT typically shows bone destruction with a soft tissue mass; sclerosis is seen in up to 75% of cases, though the classic sunburst periosteal reaction is often absent. MRI reveals low T1 and intermediate T2 signals, influenced by the tumor’s mineral content, and is effective for assessing intramedullary and extraosseous spread [Articol Cristi]. FDG uptake on PET/CT varies with histological grade, with high-grade sarcomas exhibiting more significant FDG accumulation [45].

Sinonasal rhabdomyosarcoma often involves lymph nodes and metastasizes to the lungs and bones [40]. On CT, it appears homogeneous with bone remodeling and destruction. MRI shows isointense T1 and hyperintense T2 signals with moderate to substantial enhancement [30]. While its imaging features, such as homogeneity and skull involvement, are not specific, a lower ADC value can help differentiate it from squamous cell carcinoma, reflecting the tumor’s undifferentiated microstructure [46,47].

Juvenile nasal angiofibroma, a highly vascular malformation in young males (15–25 years), typically presents with epistaxis and unilateral nasal obstruction [44]. CT and MRI reveal posterior maxillary antrum bending, pterygopalatine fossa enlargement with bone erosion, and avid enhancement [48]. MRI shows intermediate T1 and intermediate-high T2 signals, with flow voids confirming their vascular nature [30].

Inverted papilloma is a locally aggressive benign tumor often originating from the lateral nasal wall near the middle meatus, sometimes extending to the maxillary antrum [49]. CT reveals a soft tissue mass, occasionally with intratumoral calcifications and focal hyperostosis at the attachment site. MRI shows a convoluted cerebriform pattern on post-contrast T1WI [50], with hypointense epithelium and hyperintense edematous stroma on T2WI [51]. Coexistent squamous cell carcinoma, present in 5–7% of cases, is suggested by necrosis, bone destruction, or loss of cerebriform enhancement. 18F-FDG PET/CT can aid in identifying malignancy through focal increased FDG uptake [52].

Sinonasal schwannomas are benign, slow-growing nerve sheath tumors, typically appearing as well-circumscribed, ovoid soft tissue masses. Larger lesions may show cystic degeneration. On CT, subtle hyperdense areas reflect Antoni A tissue, while adjacent bone remodeling without destruction is paramount [30]. MRI reveals intermediate T1 and variable T2 signals, with contrast enhancement occasionally displaying a characteristic whorled pattern [53].

Osteoma, the most common benign sinonasal tumor, often occurs in the frontal sinus and is usually an incidental finding. On CT, cortical osteomas appear homogeneously dense, while cancellous osteomas may display heterogeneous density or a “ground-glass” pattern, sometimes resembling fibrous dysplasia. MRI typically shows low-to-intermediate signal intensity with no enhancement of the osseous contents, though fibrous tissue may enhance post-contrast [30]. Diagnosis is often aided by patient history or prior imaging studies.

Fibrous dysplasia is a fibro-osseous disorder commonly affecting craniofacial bones [30]. On CT, it may present as heterogeneous osseous and fibrous tissue or exhibit a classic “ground-glass” appearance [54]. MRI reveals variable signals, typically hypointense on T1 and T2, with intense post-contrast enhancement [55]. On 18F-FDG PET/CT, fibrous tissue may show hypermetabolic activity.

Inflammatory myofibroblastic tumor, a rare sinonasal lesion incidentally diagnosed, often mimics malignant tumors like SCC [56]. CT shows bone erosion with sclerosis, unlike malignant tumors exhibiting erosion and destruction. MRI reveals hypointense signals on T1 and T2, with substantial gadolinium enhancement [57]. Differentiating features in imaging are crucial for diagnosis.

Sinonasal hemangiopericytomas often originate from the nasal septum and turbinates, blending with surrounding structures due to substantial enhancement on contrast imaging [58]. CT may reveal a “soap bubble” or “honeycomb” appearance. MRI shows hypointense T1, hyperintense T2 signals, and avid enhancement, with intratumoral flow voids as a characteristic finding [59].

Histopathological diagnosis is confirmed through biopsy and immunohistochemical analysis, where myoglobin, Myo D1, and myogenin are specific markers for RMS [60].

For instance, Torres-Peña et al. documented two cases of alveolar RMS originating in the nasal cavity with orbital extension, emphasizing the aggressive nature of these tumors in adults and the importance of considering RMS in differential diagnoses [23,61]. Similarly, a review by Ahmed and Tsokos noted that RMS in the sinonasal region presents unique challenges, particularly due to the prevalence of the alveolar subtype and the poor prognosis associated with these tumors [26].

Recent studies have also identified characteristic imaging features of sinonasal RMS [62]. Zeng et al. analyzed MRI findings in eleven cases, noting bone destruction and multifocal sinus involvement [63]. This underscores the importance of advanced imaging techniques in diagnosing RMS.

In terms of differential diagnosis, we should include benign tumors such as papilloma, fibromyoma, chondroma, adenoma, glioma or neuroblastoma, and angiofibroma. The macroscopic aspect of the tumor, rapid growth (within a few months), and imagistic (CT/CBCT), which show the invasion of the neighboring structures, exclude most of the above [64]. Out of the malignant tumors of the nasal fossa in the child, lymphoma is the most common.

Knowing that lymphoma responds to chemotherapy, it can be put up for discussion whether it was necessary to just perform the biopsy without removing the mass to establish the right course of treatment.

## 5. RMS Treatment Options and Survival Rates

The TNM staging of RMS is crucial before treatment and is determined by tumor size and location, local invasion, lymph node involvement, and metastasis [65]. Metastatic RMS presents significant challenges due to its resistance to standard treatments and its ability to evade the body’s natural immune responses, necessitating the exploration of innovative therapeutic strategies [66]. Risk assessment is essential to tailor treatment intensity based on tumor location, presence of residual tumor post-resection, histological type, lymph node involvement, and metastasis [67,68].

For over three decades, the standard treatment protocol for RMS in Europe has consisted of a six-month chemotherapy regimen. The European Pediatric Soft Tissue Sarcoma Study Group (EpSSG) has evaluated whether extending treatment with maintenance chemotherapy could improve outcomes [69].

Management of RMS in the nasal cavity and paranasal sinuses requires a multimodal approach that typically includes surgery, chemotherapy, and radiation therapy. Historically, the role of surgery has been limited due to anatomical challenges and the significant role of systemic therapy. However, recent studies indicate that surgical intervention may improve outcomes, particularly in localized cases [25,26,63].

Surgical removal of the tumor and chemotherapy or a combination of both remains the mainstay treatment. A complete resection is achieved by resecting the tumor along with a 0.5 cm rim of normal tissue around it [70,71].

Kana et al. explored the outcomes of surgical management in patients with sinonasal RMS, highlighting that those who underwent surgical resection had improved distant metastasis-free survival compared to those who did not [25]. Moreover, Nayak et al. reported a rare case of alveolar RMS in the paranasal sinus with cervical metastasis, managed through a combination of surgery and chemoradiotherapy [24].

The role of chemotherapy is paramount, particularly in advanced cases. Chemotherapeutic regimens are often tailored based on histological subtype and clinical staging [17,18]. In children and adolescents, Machavoine et al. emphasized that younger patients and those with certain molecular features had better outcomes, indicating the importance of personalized treatment strategies [27].

Taking into account certain aspects like tumor location, stage, and age of the patient, the treatment of RMS is multimodal and individualized. It can be surgical, radiological, and chemotherapeutic.

Radiotherapy is reserved for patients who develop recurrence following completion of initial treatment. The duration of the chemotherapy is determined by the stage of the disease at the time of presentation [71].

### 5.1. Surgical Approach

Management of RMS involves a multimodal approach tailored to the individual patient, considering tumor location, stage, and age. Surgical excision is the first step, categorized into four clinical groups based on residual disease:

- group 1—The tumor is completely removed;
- group 2—Microscopic residual tumor tissue, invasive loco-regional ganglia, or both;
- group 3—High residual tumor tissue;
- group 4—Distant metastases.

Surgical treatment of RMS depends on the tumor site—It is advised to completely remove the mass if it is possible without causing lesions to impede the function of surrounding organs or disfiguring the patient [25].

Tumor excision is recommended even if there are metastases.

Under ideal conditions, RMS resection should be made 2 cm from the edge of the tumor. In the case of tumors with head localization and especially in children, this limit cannot be met, and resection is recommended at 0.5 cm from the tumor [21]. The resection margins should be noted and sent for histopathological examination in order to have precise evidence of areas where the tumor was not completely resected. They must be marked with titanium clips for targeted irradiation and possibly for reintervention.

Kainhofer et al. evaluated the prognostic significance of margin status in soft tissue sarcoma (STS) patients undergoing curative surgery [72]. Their findings indicated that margin status is an independent factor influencing local recurrence (LR). Specifically, local control rates were higher in cases with a minimal resection margin of 1 mm or more (classified as R0 by UICC standards) compared to margins less than 1 mm (classified as R0 under the R-classification) [73]. Outcomes were notably better for patients who underwent “complete primary resection” compared to those with “incomplete surgery” [74,75,76].

While a 5 mm surgical margin is often considered an optimal target, achieving such margins may not be feasible or necessary in all anatomical regions or in pediatric patients [73].

The ability to perform en bloc resections with wide margins is highly dependent on the tumor’s location, as well as the patient’s age and size. In anatomically challenging areas, such as the head and neck, achieving completely negative microscopic margins remains particularly difficult [77].

The surgical stage, when indicated, plays a vital role in treatment stratification, local control, and outcome [42]. The surgeon is assessing the risk of the treatment, he is monitoring the tumor growth and site aspect, and implicitly he contributes to the outcome of the treatment.

Within head and neck localizations, ARMS generally originates within the inferior orbit. For all head and neck RMS, a biopsy is required for diagnostic confirmation. Resection may be limited by the inability to obtain an adequate margin; therefore, the success of resection is heavily dependent on location and resection possibilities [78].

### 5.2. Radiotherapy Treatment

The patients with post-op residual macroscopic tumor tissue will undergo postoperative radiotherapy. Radiotherapy alone is not capable of destroying macroscopic lesions, while moderate-dose irradiation reduces the risk of local–regional relapse.

Concerns about the long-term toxicity of radiotherapy in children complicate its acceptance by caregivers. The prognosis for children with residual disease is poor without radiotherapy. Pappo [79] estimates a 20% 5-year survival rate in patients with embryonal RMS of group II–III who relapsed and 3% in patients of group III–IV who have relapsed in cases of alveolar RMS. Unfortunately, radiotherapy is not performed in over 40% of patients with RMS [79].

Irradiation will need to be individualized and optimized considering the tumor site and age. The radiation dose is different from author to author. According to risk factors, the American School divides patients into three groups:

- Group I—36 Gy;
- Group II—41.4 Gy;
- Group III—50.4 Gy after lesion extension [8].

The European School applies lower doses of irradiation depending on tumor histology [8]. In patients with microscopic disease, it is intended that in the future, the optimal dose and volume for the affected area should be standardized according to histology, site, and age [80,81].

Proton radiotherapy has been used with good results in a study between 1987 and 2014 on 55 patients, 35 males and 20 females, with a mean age of 5 years and a 1 to 2-year overall survival of 91.9% [82]. Another possibility is brachytherapy [83].

PET-MRI is used to study metabolic imaging using both 2-deoxy-[18 F]-fluoro-D-glucose [18 F] FDG to specify tumor size and activity [84,85].

Numerous studies have found that FDG-PET is a sensitive and specific tool in the clinical determination of disease extension in childhood sarcomas, especially when combined with CT [78,86,87].

Extension of restricted diffusion in RMS in children and hypermetabolic overlap with restricted MRI/PET diffusion demonstrated their prognostic value, indicating an unfavorable prognosis [61,88].

### 5.3. Chemotherapy Treatment

Standard medical treatment consists of vincristine-based chemotherapy, actinomycin D, and cyclophosphamide [11].

The duration of chemotherapy and dosing of cyclophosphamide is assessed according to the risk of the disease and the response to the treatment, thus limiting the toxicity of the treatment [71].

New therapeutic agents, as well as molecular therapies, are being tested in high-risk patients [89,90].

For the European Scaffolding Group of soft tissue (EpSSG), the standard treatment for RMS is ifosfamide, vincristine, and actinomycin-D [91,92].

Ifosfamide is preferred to cyclophosphamide because it is associated with lower gonadal toxicity.

In the European treatment guidelines, the following three chemotherapy courses are evaluated [93] as follows:

- Complete response—No measurable tumor;
- Good response—Reducing tumor volume by 2/3 compared to the initial volume;
- Poor response—Reduction of tumor volume ˃1/3 and ˂2/3 from the initial volume;
- Objective response—Reduction of tumor volume ˃1/3 compared to an initial volume;
- Progressive disease—The Volume increases ˃1/3 or new lesions appear.

The purpose of this assessment is to increase chemotherapy in children whose tumor reduction is below 50% [93]. According to US protocols [11], intensification of chemotherapy, according to the data provided by this guide, does not correlate with prognosis. Higher-than-needed doses of chemotherapy can have adverse effects, some also located in the orofacial region [94].

Studies with other chemotherapeutic agents such as doxorubicin [95], ifosfamide with or without etoposide [95], or topotecan [96] do not improve the progression of patients with medium-grade RMS.

Arndt [89], in a recent article, discusses the experience of 50 years of study of RMS on both sides of the Atlantic:

- It is desirable to decrease or omit the alkylating therapy;
- Doxorubicin does not improve prognosis in those at high risk;
- high dose chemotherapy and stem cells do not improve prognosis in those at high risk;
- In the USA, vincristine, actinomycin, and cyclophosphamide remain an important part of the therapy;
- In Europe, the alkylating agent is ifosfamide;
- High-risk patients with the worst prognosis have not improved their prognosis against the 1972 trial;
- Increasing the dose received to be aggressive and decreasing the risk of relapse should be considered, provided it is not possible to identify a priori children with a high risk of relapse.

### 5.4. Survival Rates

Several factors can influence the survival rates of patients with RMS, such as age at diagnosis, tumor subtype and size, primary site, disease stage and clinical group, and treatment methods. The advances in therapy regimens over the past years have raised the overall 5-year survival rate in pediatric patients to over 70%. Still, the numbers remain low for high-risk RMS and recurrent disease [97]. Furthermore, the percentages published by the Intergroup Rhabdomyosarcoma Study Group (IRSG) based on clinical trials can differ from those from population-based cancer registries due to restrictive inclusion criteria [98].

As previously mentioned, the primary treatment modality is complete surgical resection of the tumor. The IRSG/COG (Intergroup Rhabdomyosarcoma Study Group/Children’s Oncology Group) stated that operability was the most significant factor affecting patient survival, with completely resected localized tumors associated with a survival rate of 87% [99].

Tumors less than 5 cm, with no evidence of regional lymph node involvement or distant metastases, have a better prognosis [100]. For a tumor 10 cm in diameter, the survival rate is reduced. Because most RMS lesions are not spherical but irregular in shape, there was a debate regarding the informative value of tumor size vs. tumor volume evaluation for a patient’s prognosis. Ferrari et al. addressed this issue and concluded that assessing tumor size from one or three dimensions provided a similar prognostic value [101].

Adjuvant chemotherapy and radiotherapy can be recommended to enhance outcomes. Yang et al. designed a prognostic nomogram based on the records of 1679 RMS pediatric patients to predict patient outcomes. They observed that surgery has significantly increased the survival rate (*p*  <  0.001), and radiotherapy was also significantly associated with a better prognosis, but to a much lesser extent [98]. The advanced radiotherapy techniques available (protons, brachytherapy, and rotational intensity-modulated radiotherapy) have contributed to significant improvements in outcomes, as reported by the European Paediatric Soft Tissue Sarcoma Group (EpSSG). However, there are still cases where this approach is ineffective [102].

## 6. Our Experience—Case Report

CR, an 8-year-old male from a rural area, presents with predominantly left nasal obstruction, purulent rhinorrhea, recurrent epistaxis, and headache. He is under endocrinology surveillance for a chronic adrenogenital syndrome, treated with prednisone (4 mg/day). Symptoms began in 2017, leading to hospitalization in January 2018 at the Pediatric Hospital in Iasi. During hospitalization, antibiotic therapy (Xifia 5 mL every 12 h), mucolytics, and intravenous calcium were initiated. Due to persistent symptoms, a CT scan was performed, and an ENT consultation was sought. Correlating the ENT clinical examination with symptomatology and CT findings, the patient was referred to the ENT department of the Clinical Rehabilitation Hospital for further evaluation and surgical intervention.

Clinical examination revealed a mass in the left nasal fossa that completely obstructed the cavity. The tumor appeared violet, was covered in purulent secretions, and exhibited adherence with bleeding upon palpation. The right nasal fossa contained muco-purulent secretions but no mucosal abnormalities, with a posterior view of the mass occupying the entire opening. Nasal and sinus endoscopy highlights the same issues: a violet mass covered with secretions and puss blocking the left nasal fossa, without invasion in the nasal fossa structures (septum, floor, or lateral wall), and without ulcerations on the mucosal surfaces. Access to the rinopharynx is difficult and reveals a mass that completely obstructs the choane with apparent origin from the superior wall of the rinopharynx. The overlying mucosa with smooth, without ulcerations, but with multiple pink-reddish fleshy polyp-like hypertrophy.

CT imaging revealed a soft tissue mass measuring 72 mm × 42 mm × 22 mm with reduced contrast enhancement, extending from the left nasal fossa into the nasopharynx, posterior wall, and ethmoid cells on the left side, as well as into the left maxillary sinus and posterior part of the right nasal fossa, ultimately occupying the sphenoid sinus. The nasal septum appeared discontinuous in the posterior third. A latero-cervical ultrasound excluded lymph node involvement. CBC results were normal, while coagulation tests indicated minor alterations.

To obtain a biopsy of the lesion, surgical excision of the bulk of the tumor occupying the nasal fossa and nasopharynx was necessary to detect and reach the insertion point. A biopsy sample was sent for extemporaneous examination. While awaiting the result, the paranasal sinuses were accessed, and maxillary antrostomy, ethmoidectomy, sphenoidectomy, and excision of the abnormal-looking tissue from the inferior part of the sphenoid floor were performed. The posterior part of the nasal septum was found to be soft, likely due to compression from the tumor. However, no visible mucosal involvement was observed, possibly because the posterior bony part had thinned under pressure.

The extemporaneous examination suggested a high-grade non-Hodgkin lymphoma (B.A.P. 27855/9. January 2018).

The surgical interventions were deemed necessary for several reasons: obtaining a biopsy close to the insertion point required intubation of the patient; regardless of the biopsy findings, surgical management of the polisinusitis was essential; and excision of the abnormal tissue provided benefits for both immediate and long-term patient outcomes.

**Figure 1:**
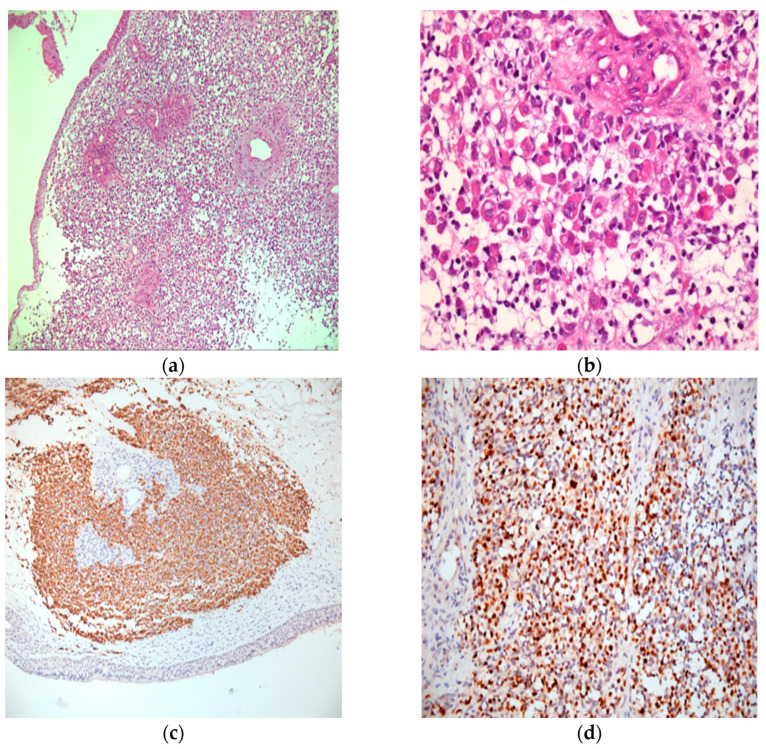
Histopathology aspect with different colors and magnification (**a**) RMS, HE, ×100, overview, rhabdomyoblastic proliferation with solid pattern, mucosa on a slope (**b**) RMS alveolar, HE, ×400, detail rhabdomyoblast; (**c**) Desmin, ×100, positive diffuse in the tumor; (**d**) Myogenin, ×200, diffuse positive in most cells (nuclear marker).

All excised tissue was sent for histopathological exam. Further immunohistochemical analysis was conducted to confirm and differentiate the tumor subtype from other morphologically similar malignancies (Figure 1). Although the initial findings suggested non-Hodgkin lymphoma, subsequent tests ruled it out and established the definitive diagnosis of high-grade alveolar rhabdomyosarcoma (RMS) (ICD-O 8920/3).

Chemotherapy was initiated, and following nine rounds, a post-therapeutic MRI indicated minimal inflammatory lesions in the left maxillary sinus mucosa and several ethmoid cells bilaterally.

**Figure 2:**
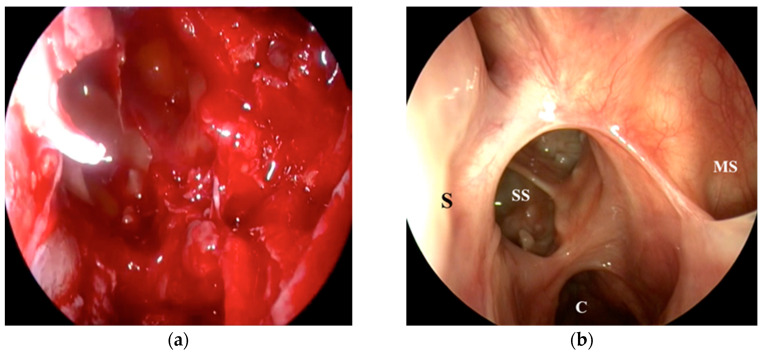
Endoscopic view at the moment of surgery and at the 6 years follow-up. (**a**) Intraoperative endoscopic image of left nasal fossae and sphenoid sinus; (**b**) follow-up visit at 6 years show cavum (C), septum (S), sphenoid sinus (SS), and maxillary sinus (MS).

Post-chemotherapeutic rhinosinusal endoscopy only reveals the presence of mucus in the maxillary sinus and discrete diffuse edema of the nasal mucosa without any local tumor recurrence. Follow-up evaluations were conducted annually, with the most recent assessment in 2024 (6 years after treatment) showing no evidence of tumor recurrence in the rhinopharynx and paranasal sinuses. Endoscopic examination revealed no scarring, stenosis, or mucosal retention (Figure 2). PET CT at 5 years showed no signs of disease persistence.

In this case, the necessity for tumor ablation is underscored by several factors. The tumor caused nasal obstruction and blocked the sinuses that were already suffering, and for the biopsy to be conclusive under conditions of such a large and brittle mass, it was necessary to remove the tumor from its insertion site.

The initial result of the histopathological exam was lymphoma, due to which chemotherapy would have been recommended.

Further histochemical examination of the tumor established the diagnosis of RMS.

In the case we presented, the treatment consisted of three rounds of vincristine, actinomycin-D, and cyclophosphamide.

Endoscopic evaluation one month after completing three rounds of chemotherapy could be considered positive regarding the fact that there were no macroscopic signs of tumor recurrence.

However, given that an alveolar RMS has a worse prognosis compared to the embryonal one, radiation therapy is recommended with reassessment after a month. A 3-month basis endoscopic examination and MRI for further follow-up is advised.

The prognosis for this patient remains reserved due to tumor size (tumors larger than 5 cm with a poor prognosis) and histopathological type (alveolar RMS). Recent follow-up has shown disease-free outcomes at 6 years of check-up.

Diagnosis is determined by anatomopathological examination and will be followed by radical surgery, radio, and chemotherapy

## 7. Conclusions

Alveolar RMS is a rare tumor encountered in the pediatric population. High suspicion is needed in diagnosing this condition in the early stages.

RMS in children should be taken into account whenever a patient is referred for a vegetative tumor in the rhinopharynx or nasal fossa.

Prognosis is reserved and depends on correct evaluation and early treatment. Rigorous follow-up is mandatory and will discover reoccurrences earlier allowing better treatment prognosis.

RMS presents significant challenges in pediatric management, necessitating a coordinated approach involving surgical intervention, radiotherapy, and chemotherapy. Individualized treatment plans are essential to optimize outcomes while minimizing long-term toxicity. Ongoing surveillance through imaging and endoscopic evaluation is crucial for early detection of recurrence, given the complex nature of this malignancy.
